# A Public Health Analysis on Gaps in Disease Monitoring and Opportunities for Improved Care for the Management of Hepatitis B and C

**DOI:** 10.7759/cureus.2077

**Published:** 2018-01-16

**Authors:** Faisal Akhtar, Sabah Rehman

**Affiliations:** 1 Neurology, Ochsner Health System; 2 Biochemistry, Shifa College Of Medicine

**Keywords:** hepatitis c, hepatitis b, hcv, hbv, public health, public health analysis, improved care, disease monitoring, disease management

## Abstract

Hepatitis B and Hepatitis C have been major disease-causing agents among humans since they were discovered in the 1960s. Both cause jaundice-like symptoms initially but their prognosis and treatment are somehow different and depend upon many demographic details, such as the age and susceptibility of the patients and any other comorbid conditions. They clinically present primarily with hepatitis and can have many adverse effects or even be life-threatening at times, if not treated properly. However, their epidemiological background and findings in terms of morbidity, mortality, and case fatality rates are different. The disease burden, impact on the healthcare system, and prevention of the two diseases are quite different. The treatment and management options along with the prevention and control measures share unique strategies for handling the two diseases. The purpose of this review is to highlight the gaps in disease monitoring and to find ways and opportunities that can lead to improved care and better management of Hepatitis B and C locally and globally. Online databases were searched and peer-reviewed articles were selected. Key issues identified were lack of education globally in resource-limited settings, leading to a decreased understanding of the potential hazards associated with needle sharing and lack of access to healthcare because of a lack of insurance. The failure of compliance with vaccination leads to an increase in mother-to-child transmission (MTCT)-related infections. Increased global travel demands a systematic program in most immigrant-receiving countries to screen for hepatitis B virus (HBV)/hepatitis c virus (HCV) infections. Delayed U.S. Food and Drug Administration (FDA) licensing for new drugs hampers the treatment of chronic Hepatitis-B (CHB) among children. With the advancement in science, an effective vaccine against HCV will definitely help in eradicating the infection.

## Introduction and background

Chronic Hepatitis B and C infections are prevalent diseases and add a substantial burden to healthcare systems, with both accounting for global morbidity and mortality of approximately one million deaths that are attributable to them or/and their sequelae [1].

Hepatitis B

The clinical features of the hepatitis B virus (HBV) infection ranges from asymptomatic infections to jaundice following a flu-like illness. Persons with acute HBV may develop serum sickness-like illness and chronic infection, which is associated with glomerulonephritis or vasculitis [2].

The HBV infection in infants and children rarely results in jaundice, and such young patients usually remain completely asymptomatic. In infants born to a mother who is a hepatitis B e-antigen (HBeAg)-positive carrier, the risk of infection with chronic carriage (defined as carriage for more than one year) is approximately 80% if the HBV vaccination is not initiated soon after birth. The risk of chronic infection decreases with increasing age. By age six, the chronic infection occurs in 5% to 10% of individuals and in 1% to 5% of adolescents and adults. HIV positive patients, patients on dialysis, oncology and transplant patients, and those who are immunosuppressed have a high risk of developing chronic HBV.

Disease Burden

HBV infects over two billion people worldwide, with approximately 360 million chronically infected. It results in substantial morbidity and mortality, with an estimated 600,000 deaths per year [3].

In prospective studies in Taiwan, 25% of persons infected during their childhood and who became carriers of HBV developed hepatocellular carcinoma (HCC) during their lifetime. Nazzal Z et al. conducted a case-control study on 100 cases and 100 controls for the risk factors of hepatitis B transmission in northern Palestine. They performed a univariate analysis and logistic regression model to examine the probable risk factors of hepatitis B infection. Nazzal Z et al. found out that that the cases had a history of blood transfusion, dental visits, hospitalization, sharing shaving equipment, intravenous (IV) drug use, or living abroad as compared to the controls [4].

Hepatitis C

Common risk factors like medical procedures, lifestyle-associated risk factors, IV drug abuse, and tattoos/piercings were also noticed [5]. Hepatitis C virus (HCV) is transmitted parenterally, thus putting healthcare professionals at risk of infection, especially dental healthcare workers who need to recognize the symptoms of the infection that may be present in the oral cavities of infected individuals [6].

Transmission of the hepatitis C virus is of critical importance, as it is a key step to stop the virus from spreading by stopping the route of transmission. Primarily, HCV is transmitted via contact with the blood of infected individuals; other forms of contact with human blood or/and secretions are also likely to transmit the virus, but at a lower frequency [7].

The hepatitis C virus infects men more than women, and after the initial infection, women are more likely to clear the virus than men, with slower rates of liver disease progression as well. However, the disease progression changes over time with postmenopausal women having increased rates of fibrosis due to the lost protective effects of estrogen; thus, the disease burden from HCV is mostly predominant in men [8].

Disease Burden

HCV infects more than 185 million individuals worldwide. Twenty percent of patients chronically infected with HCV progress to cirrhosis [9]. Approximately 10,000 persons die of the HCV infection each year in the United States [2], increasing the burden on healthcare systems. The HBV infection is a major public health problem worldwide, with roughly 30% of the world's population showing serological evidence of current or past infection.

## Review

Goals and objectives

Because of the sequelae of chronic liver diseases, including cirrhosis and hepatocellular carcinoma (HCC), infections like HBV and HCV are still major causes of morbidity and mortality globally [10]. In sub-Saharan Africa, HBV and HCV transmission via blood transfusions is very high and reducing the risk of transfusion-transmitted human infection is a priority of international aid organizations recently. The efforts of the U.S. President’s plan for AIDS relief (PEPFAR), the World Health Organization, the global fund to combat HIV/AIDS, malaria, and tuberculosis (TB), and HBV- and HCV-related surveillance programs to evaluate these two diseases in 38 sub-Saharan African countries has increased the screening of at least 95% blood donations for HBV and HCV, decreasing the national prevalence of HBV and HCV [10].

Although it is understood that hepatitis B and hepatitis C are common predisposing factors leading to cirrhosis and liver cancer, therapies for hepatitis B suppress viral replication and improve morbidity and mortality; hepatitis C is a treatable disease, so if it is diagnosed early and treated, it can lead to viral eradication [11].

The goal of this review is to highlight the gaps in the management of hepatitis B and C, from the disease evaluation phase to monitoring and improved care. The attempt is to highlight the burden of the disease and the limitations in improving patient care, both nationally and internationally. Addressing these key issues can have a significant impact on the disease prevalence and reduce the burden on healthcare by further diminishing the impact caused by the co-morbid conditions associated with these diseases.

Materials and methods

The methods used for gathering research for this analysis included extensive searching on the Tulane University Howard Tilton Library Internet database. Databases such as PubMed, Google Scholar, Public Health Grey literature, ClinicalKey, and Embase were searched for peer-reviewed articles. The abstract summaries of all the articles were read and then the relevant ones were selected and read in detail. Articles older than 2014 and those not in English were not included in the final selection.

Results

A study by Islam et al. found that more than half of the study participants had inadequate knowledge of liver disease, with higher percentages of people with very limited knowledge of HBV and HCV in particular. They also found out that the mean knowledge score was increased among patients with higher education, a history of HCV screening, and low alcohol consumption [12].

There is an estimate that around 90% of individuals infected with HCV worldwide reside in resource-limited settings [13]. One of the key challenges faced by the world today in controlling and preventing hepatitis, such as HBV, is the widespread use of infected, shared needles among drug users, which is, by far, the most important element in the transmission of the disease. A research was conducted in India [13] among 14481 people who injected drugs of different kinds, and samples were collected from 15 cities across India from January 2, 2013, to December 19, 2013. HCV prevalence was estimated by the presence of anti-HCV antibodies. The study showed that the median age of the participants was 30 years, with 13608 being men. The weighted HCV prevalence was 5777 (37.2%). It was higher among the community with a lifetime injection frequency, alcohol use, reported needle sharing, and other diseases like HIV positive. Among these people, 440 were aware of their status, 225 had seen a doctor for their HCV, and 79 had taken HCV treatment, while 18 had undetectable HCV ribonucleic RNA. From such examples, it can be deduced that an awareness of HCV positive status was significantly associated with higher education, HIV testing history, knowledge of HIV positive status, and community involvement in therapy coverage. Such communities can be an alarming sign and can act as a neglected epidemic.

The morbidity and mortality rate of HCV is higher among people who inject drugs (PWID) because of the other, associated risk factors and comorbidities. Recently, data were derived from a long-term cohort of PWID in Vancouver, Canada [14] in order to evaluate the HCV incidence and associated risk factors among PWID recruited from 1996-2012. It was seen that the overall density of HCV incidence declined significantly from 25/100 in 1996-1999 to 6/100 in 2000-2005, and 3.1/100 in 2006-2012. Among PWID, a downward trend in the incidence density was noticed, with 27.9/100 in 1996-1999, to 7.5/100 in 2000-2005, and 4.9/100 in 2006-2012. Thus, the study concluded that the HCV incidence declined dramatically among PWID, however, improved public health strategies to prevent and treat HCV are still urgently required to reduce the HCV-associated morbidity and mortality [14].

No more than one in five individuals with jaundice-like symptoms will seek medical care, so most of the times, an HCV infection is unnoticed [2]. In other cases, it becomes difficult to distinguish from other hepatitis viruses. Malaise is a clinical symptom that may persist for 10-50 years among 60%-85% individuals with persistent viremia (can show elevated liver enzymes), after an acute infection of HCV, but is still not an indicator to be diagnosed and consulted for hepatitis C.

Due to the increased number of travelers among different countries and continents, immigrants have increased mortality from HCC as compared to the host populations, primarily due to an undetected chronic HBV infection, as there are no programs for the evaluation of their disease status so far. This can also be a potential threat to the host population [15].

MTCT is the most important source of new infections in endemic areas, but even in low endemicity areas, over 1/3^rd^ of infections can still be attributed to this route. Although a very effective HBV vaccine is available, even with full compliance, failure can be seen in highly viremic mothers who are positive for HBeAg.

The vertical transmission of HBV is most important in endemic areas, but, still, 3%-10% of all the cases of HBV is due to the mother-to-child transmission of HCV, and it is still the leading route of infection among children [16].

Burden on the healthcare system in the US

It is estimated that around six billion ambulatory care visits occurred in the US from 2006 to 2010, of which an estimated 25.8 million (0.43%) were chronic liver disease (CLD) related. Among adults aged 45–64 years, Medicaid and Medicare recipients were 3.9 and 2.3 times more likely to have a CLD-related ambulatory visit than those with private insurance, respectively. In the United States, from 2006 to 2010, an estimated 49.6% of all CLD-related ambulatory visits were attributed solely to viral hepatitis B and C diagnoses. Viral hepatitis is an important etiology of CLD in the US, with hepatitis B and C contributing approximately one-half of the CLD burden [17].

An estimated 1% to 1.9% of North Americans are infected with HCV [18]. Despite prevention and vaccination, the HBV infection is still a major health problem that most commonly affects the immigrant population and men [19].

A chronic hepatitis cohort study, investigating baseline demographic, clinical, and mortality data from the electronic health records of > 220 chronic HBV and 8800 chronic HCV patients from four integrated healthcare systems, indicated the substantial burden of chronic viral hepatitis in the US. It assessed the clinical impact of chronic viral hepatitis in the US. More than 1.6 million adult patients’ records were seen from January 2006 through December 2010, showing that on the basis of baseline demographic, hospitalization, and mortality data, the study highlighted the substantial US health burden from chronic viral hepatitis, particularly among persons born during 1945–1964 [20].

Burman BE et al. studied and highlighted the gaps in disease monitoring and opportunities for improved care and the management of hepatitis B among vulnerable populations. They suggested that in certain US populations, HBV is prevalent and regular monitoring is critical to reduce the morbidity and mortality associated with the HBV infection [21].

Discussion

Hepatitis C

Chronic HCV infection is a major public health burden associated with significant morbidity and mortality. Recently, teleprevir and boceprevir have been approved for use with interferon and ribavirin in chronic HCV, with an increase in viral eradication but associated with increased adverse events as well, thus a decrease in clinical tolerance. Direct-acting antiviral and host-targeting agents are newer classes of antiviral agents that offer drug combinations without the need for interferon, with better response rates and a shorter duration of treatment [22].

By the end of 2013, treatments of HCV took a great step with the approval of drugs like simeprevir and sofosbuvir. These drugs can be used in different combination regimens with ribavirin for treating different genotypes. This gives clinicians and patients the opportunity to discuss and select from current treatment options or await upcoming regimens [23]. Oral antiviral agents, simeprevir and sofosbuvir, have already been approved and are now available for the treatment of patients with chronic HCV. Other antiviral agents were available during 2014. With these drugs, it is now possible to cure chronic HCV in the vast majority of patients with chronic HCV and in many patients without interferon [24].

A literature search was conducted from January 1, 2009, till May 30, 2014, evaluating the treatment of HCV, with 41 studies involving 19063 adult patients. HCV treatment recommendations were formulated, suggesting that HCV genotype-1 represents 60%-75% of HCV infections in the US and is more difficult to cure than genotype-2 or genotype-3, and type-1 patients should receive treatment with sofosbuvir + pegylated interferon + ribavirin whereas patients with type-2 and type-3 should receive therapy with sofobuvir + ribavirin alone. The result concluded that newer, short-duration, simpler therapies result in higher SVR rates for HCV-infected patients [9].

A recent Japanese study investigated the evolution of simeprevir-resistant variants in patients at Toranomon Hospital infected with HCV genotype-1b and who were given triple therapy of simeprevir/PEG-IFN/ribavirin. The study concluded that the emergence of simeprevir-resistant variants after the start of treatment could not be predicted at the baseline, and the majority of de novo resistant variants become undetectable over time [25].

No more than one in five individuals with jaundice-like symptoms will seek medical care, so, most of the times, an HCV infection is unnoticed [2]. In other cases, it becomes difficult to distinguish from other hepatitis viruses. Malaise is a clinical symptom that may persist for 10-50 years among 60%-85% individuals with persistent viremia (can show elevated liver enzymes), after an acute infection of HCV, but it is still not an indicator to be diagnosed and consulted for hepatitis C.

Injection drug use is the major cause of the transmission of the hepatitis C virus, and studies have suggested that the use of the opioid agonist therapy can reduce the incidence of the HCV infection among injection drug users. An observational cohort study recruited young adult (younger than 30 years) injection drug users who were negative for the anti-HCV antibody or/and HCV RNA. The study was conducted from January 03, 2000, till August 21, 2013, with quarterly interviews and blood sampling [26]. Study subjects were divided into groups and the opioid agonist group was compared with the non-opioid agonist form of treatment. The results showed that those young adult injection drug users who received opioid therapy were associated with a lower incidence of HCV infection. So, they concluded that treatment with methadone or buprenorphine for opioid use disorders can be an effective strategy in order to prevent HCV spread among young injection drug users.

To increase the efficacy of HCV treatment, future regimens should incorporate multiple direct-acting antiviral drugs. The HCV NS5A protein was expressed and purified. Aptamers for NS5A may be used to understand the mechanisms of virus replication and assembly and served as potential therapeutic agents for hepatitis C [27].

With increased screening for HCV, as recommended by recent Centers for Disease Control and Prevention (CDC) guidelines, and with the availability of newer therapies that may lead to the treatment of many more chronic HCV infections, the prevalence as well as case fatality rates of the disease are decreasing [9].

Hepatitis B

Entecavir is the drug of choice as the first-line treatment for treatment-naive HBV patients. With a high genetic barrier to resistance, treatment failure remains rare [28].

Telbivudine (LdT) or lamivudine (LAM) use in late pregnancy for highly viremic mothers was equally effective in reducing mother-to-child transmission (MTCT). The treatment was well-tolerated with no safety concerns identified [29].

In the United States each year, 24,000 infants are born to women who are infected with HBV and an estimated 1000 newborns acquire the infection through vertical transmission from their mother. MTCT rates of HBV can be greatly reduced if the current guidelines for screening and immunization are universally followed. The use of oral antiviral therapy in highly viremic mothers to reduce mother-to-child transmission is controversial but should be considered on a case-by-case basis [30]. Kubo A et al. conducted an observational study to study the prevention of the vertical transmission of hepatitis B and concluded that both prenatal HBV screening and postnatal prophylaxis is highly effective in preventing the vertical transmission of the disease [31].

Full compliance with the HBV vaccine schedule has also been an increasing concern since MTCT is one of the most important sources of new infections, especially among the endemic areas. However, even in low endemic areas, a significant 1/3^rd^ of the infections has been attributed to this route [4].

With newer drugs, the potential means of reducing the risk of MTCT include nucleotide/nucleoside antiviral agents, interferon in very select cases, mode of delivery and determining the optimal therapy, and preventing both obstetric and liver-related complications remains a challenge but is also an important opportunity to reduce chronic hepatitis B infections [3].

The availability of the newer drugs and therapeutic strategies is, on one hand, beneficial but, on the other hand, increases the complexity and individualizes the management of children with hepatitis, making it extremely important to educate and advise pediatricians concerning the new lines of treatment options available [1]. Although there are several guidelines currently available for the management of adult patients with HBV, unfortunately, the clinical approach to infected children is still in the primitive stage and standard interferons (IFN)-a is still the treatment of choice among children with HBV infections. The licensing of highly effective drugs for the treatment of HBV in older children and adolescents has certainly opened up new possibilities in treatment options, however, the risk of the emergence of drug-resistant strains is a public health problem and a major long-term issue even for young patients [1].

Though we now understand that the early and accurate diagnosis of cirrhosis is important for an appropriate treatment strategy to predict the prognosis of patients with chronic hepatitis-B (CHB) and a liver biopsy is still considered the reference standard for the assessment of liver fibrosis. However, there are a few limitations of this method, for example, it is invasive, associated with pain, and presents complications that can be fatal at times. Intra- and inter-observer variability also limit the accuracy of liver biopsy data [32].

Qureshi et al. studied the mother to child transmission of hepatitis B virus infection in Pakistan and evaluated if any change in immunization policy is required. Their study concluded that the current hepatitis B vaccination regimen of doses at 6, 10, and 14 weeks are not sufficiently protective and strongly urged the need for an introduction of a birth dose into the national immunization system [33].

Some of the concerns resulting in increasing disease prevalence among developing countries reside in the inadequacy of the knowledge of the population in general and knowledge of liver diseases and their presentation. A study by Islam et al. showed that the mean knowledge score was higher in patients with higher education, a history of screening, and low alcohol consumption [12].

Some of the gaps leading to increasing disease prevalence and burden on healthcare settings worldwide are resource-limited settings, lack of resources, government involvement and interest in health in general, lack of education resulting in patients reaching hospitals with terminal conditions, all of which further add to the burden [13].

Needle sharing is also one of the reasons HBV prevalence is higher among drug users, advocating resource-limited settings and lack of adequate knowledge and resource allocation in the target population [13].

In developed nations, newer cases are also reported among the immigrants, as no centralized program of evaluation has been formulated by these countries to detect (screen) the incoming population, which adds to the healthcare cost since immigrants have an increased risk of HCC due to undetected HBV infections [15]. The prevalence of hepatitis B varies greatly between different parts of the world with an increase in prevalence in recent years in the United Kingdom, apparently due to an increase in migration from areas with a high prevalence of chronic hepatitis B [34].

## Conclusions

The high burden of an HCV and HBV co-infection coupled with low access to HCV services emphasizes an urgent need to include resource-limited settings in the global HCV agenda. Although new treatments will become available worldwide in the near future, programs to improve awareness and reduce disease progression and transmission need to be scaled up without further delay. While the prevalence of a chronic HCV infection may have peaked, the disease burden continues to grow. Increased treatment uptake and efficacy combined with efforts to reduce disease transmission will help prevent advanced liver diseases and deaths. Chronic viral hepatitis B and C infections are highly prevalent and create a substantial burden on healthcare systems globally. Despite its benign course, chronic hepatitis B during childhood and adolescence develops liver cirrhosis or hepatocellular carcinoma (HCC) before adulthood. The treatment of CHB in childhood has been hampered by the long delay in licensing new drugs for pediatric use. Safe and effective antiviral therapies are available in adults, but few are labeled for use in children, and an accurate selection of who to treat and the identification of the right timing for treatment are needed to optimize response and reduce the risk of antiviral resistance. There are no systematic programs in most immigrant-receiving countries to screen for chronic HBV infection and immigrants are not routinely offered HBV vaccinations outside of the universal childhood vaccination program. Much progress has been made in the prevention of HCV transmission and in therapeutic intervention. However, even if a new wave of directly acting antivirals promise to overcome the problems of the low efficacy and adverse effects observed for the current standard of care, which include interferon-α and ribavirin, an effective vaccine would be the only means to definitively eradicate infection and to diminish the burden of HCV-related diseases at affordable costs. Although there is strong evidence that the goal of a prophylactic vaccine could be achieved, there are huge development issues that have impeded reaching this goal and that still have to be addressed.

## References

[1] El-Shabrawi M, Hassanin F (2014). Treatment of hepatitis B and C in children. Minerva Pediatr.

[2] Nelson KE, Williams CM, Graham NM (2004). Infectious Disease Epidemiology. https://books.google.com/books/about/Infectious_Disease_Epidemiology.html?id=8S84nwEACAAJ.

[3] Vodkin I, Patton H (2014). Management of hepatitis B virus infection during pregnancy. Minerva Gastroenterol Dietol.

[4] Nazzal Z, Sobuh I (2014). Risk factors of hepatitis B transmission in northern Palestine: a case - control study. BMC Res Notes.

[5] Rao H, Wei L, Lopez-Talavera JC (2014). Distribution and clinical correlates of viral and host genotypes in Chinese patients with chronic hepatitis C virus infection. J Gastroenterol Hepatol.

[6] Garbin CA, de Souza NP, de Vasconcelos RR, Garbin AJ, Villar LM (2014). Hepatitis C virus and dental health workers: an update. Oral Health Prev Dent.

[7] Rosa RS, Martinelli AL, Passos AD (2014). Risk factors for hepatitis C virus transmission in the municipality of Catanduva, state of Sao Paulo: a case-control study. Rev Soc Bras Med Trop.

[8] Baden R, Rockstroh JK, Buti M (2014). Natural history and management of hepatitis C: does sex play a role?. J Infect Dis.

[9] Kohli A, Shaffer A, Sherman A, Kottilil S (2014). Treatment of hepatitis C: a systematic review. JAMA.

[10] Apata IW, Averhoff F, Pitman J (2014). Progress toward prevention of transfusion-transmitted hepatitis B and hepatitis C infection―sub-Saharan Africa, 2000-2011. MMWR Morb Mortal Wkly Rep.

[11] Duddempudi AT, Bernstein DE (2014). Hepatitis B and C. Clin Geriatr Med.

[12] Islam N, Flores YN, Ramirez P, Bastani R, Salmeron J (2014). Hepatitis and liver disease knowledge and preventive practices among health workers in Mexico: a cross-sectional study. Int J Public Health.

[13] Solomon SS, Mehta SH, Srikrishnan AK (2015). Burden of hepatitis C virus disease and access to hepatitis C virus services in people who inject drugs in India: a cross-sectional study. Lancet Infect Dis.

[14] Grebely J, Lima VD, Marshall BD (2014). Declining incidence of hepatitis C virus infection among people who inject drugs in a Canadian setting, 1996-2012. PLoS One.

[15] Rossi C, Schwartzman K, Oxlade O, Klein MB, Greenaway C (2013). Hepatitis B screening and vaccination strategies for newly arrived adult Canadian immigrants and refugees: a cost-effectiveness analysis. PLoS One.

[16] Gentile I, Zappulo E, Buonomo AR, Borgia G (2014). Prevention of mother-to-child transmission of hepatitis B virus and hepatitis C virus. Expert Rev Anti Infect Ther.

[17] Roberts HW, Utuama OA, Klevens M, Teshale E, Hughes E, Jiles R (2014). The contribution of viral hepatitis to the burden of chronic liver disease in the United States. Am J Gastroenterol.

[18] Uhanova J, Tate RB, Tataryn DJ, Minuk GY (2013). The epidemiology of hepatitis C in a Canadian indigenous population. Can J Gastroenterol.

[19] Poves-Martinez E, del Pozo-Prieto D, Costero-Pastor B (2012). Diagnostic incidence of the presence of positive HBsAg: epidemiologic, clinical, and virological characteristics. Rev Esp Enferm Dig.

[20] Moorman AC, Gordon SC, Rupp LB (2013). Baseline characteristics and mortality among people in care for chronic viral hepatitis: the chronic hepatitis cohort study. Clin Infect Dis.

[21] Burman BE, Mukhtar NA, Toy BC (2014). Hepatitis B management in vulnerable populations: gaps in disease monitoring and opportunities for improved care. Dig Dis Sci.

[22] Lim TR, Tan BH, Mutimer DJ (2014). Evolution and emergence of a new era of antiviral treatment for chronic hepatitis C infection. Int J Antimicrob Agents.

[23] Muir AJ (2014). The rapid evolution of treatment strategies for hepatitis C. Am J Gastroenterol.

[24] Shiffman ML (2014). Hepatitis C virus therapy in the direct acting antiviral era. Curr Opin Gastroenterol.

[25] Akuta N, Suzuki F, Sezaki H (2014). Evolution of simeprevir-resistant variants over time by ultra-deep sequencing in HCV genotype 1b. J Med Virol.

[26] Tsui JI, Evans JL, Lum PJ, Hahn JA, Page K (2014). Association of opioid agonist therapy with lower incidence of hepatitis C virus infection in young adult injection drug users. JAMA Intern Med.

[27] Yu X, Gao Y, Xue Xue (2014). Inhibition of hepatitis C virus infection by NS5A-specific aptamer. Antiviral Res.

[28] Solmone M, Giombini E, Vincenti D (2014). Slow response to entecavir treatment in treatment-naive HBV patients is conditioned by immune response rather than by the presence or selection of refractory variants. Antiviral Ther.

[29] Zhang H, Pan CQ, Pang Q, Tian R, Yan M, Liu X (2014). Telbivudine or lamivudine use in late pregnancy safely reduces perinatal transmission of hepatitis B virus in real-life practice. Hepatology.

[30] Whittaker G, Herrera JL (2014). Hepatitis B in pregnancy. South Med J.

[31] Kubo A, Shlager L, Marks AR, Lakritz D, Beaumont C, Gabellini K, Corley DA (2014). Prevention of vertical transmission of hepatitis B: an observational study. Ann Intern Med.

[32] Lee S, Kim DY (2014). Non-invasive diagnosis of hepatitis B virus-related cirrhosis. World J Gastroenterol.

[33] Qureshi H, Javaid N, Alam SE, Bile KM (2014). The evidence of mother to child transmission of hepatitis B virus infection in Pakistan and the need for hepatitis B immunization policy change. J Pak Med Assoc.

[34] Oakes K (2014). Hepatitis B: prevalence and pathophysiology. Nurs Times.

